# Inhibition of RNA-binding protein HuR reduces glomerulosclerosis in experimental nephritis

**DOI:** 10.1042/CS20200193

**Published:** 2020-06-26

**Authors:** Simeng Liu, Zhimin Huang, Anna Tang, Xiaoqing Wu, Jeffrey Aube, Liang Xu, Changying Xing, Yufeng Huang

**Affiliations:** 1 Department of Internal Medicine, Division of Nephrology and Hypertension, University of Utah Health Science, Salt Lake City, UT, U.S.A.; 2 Department of Internal Medicine, Division of Nephrology, Nanjing Medical University Jiangsu Province Hospital, Nanjing, China; 3 Department of Molecular Biosciences, University of Kansas, Lawrence, KS, U.S.A.; 4 Department of Chemical Biology and Medical Chemistry, Eshelman School of Pharmacy, University of North Carolina, Chapel Hill, NC, U.S.A.

## Abstract

Recent identification of an RNA-binding protein (HuR) that regulates mRNA turnover and translation of numerous transcripts via binding to an ARE in their 3′-UTR involved in inflammation and is abnormally elevated in varied kidney diseases offers a novel target for the treatment of renal inflammation and subsequent fibrosis. Thus, we hypothesized that treatment with a selective inhibition of HuR function with a small molecule, KH-3, would down-regulate HuR-targeted proinflammatory transcripts thereby improving glomerulosclerosis in experimental nephritis, where glomerular cellular HuR is elevated. Three experimental groups included normal and diseased rats treated with or without KH-3. Disease was induced by the monoclonal anti-Thy 1.1 antibody. KH-3 was given via daily intraperitoneal injection from day 1 after disease induction to day 5 at the dose of 50 mg/kg BW/day. At day 6, diseased animals treated with KH-3 showed significant reduction in glomerular HuR levels, proteinuria, podocyte injury determined by ameliorated podocyte loss and podocin expression, glomerular staining for periodic acid-Schiff positive extracellular matrix proteins, fibronectin and collagen IV and mRNA and protein levels of profibrotic markers, compared with untreated disease rats. KH-3 treatment also reduced disease-induced increases in renal TGFβ1 and PAI-1 transcripts. Additionally, a marked increase in renal NF-κB-p65, Nox4, and glomerular macrophage cell infiltration observed in disease control group was largely reversed by KH-3 treatment. These results strongly support our hypothesis that down-regulation of HuR function with KH-3 has therapeutic potential for reversing glomerulosclerosis by reducing abundance of pro-inflammatory transcripts and related inflammation.

## Introduction

Chronic kidney disease (CKD) is characterized by persistent inflammation and progressive fibrosis including glomerulosclerosis and tubulointerstitial fibrosis, ultimately leading to end-stage renal disease (ESRD) regardless of the underlying disorder. This points to a final common pathway for ESRD. Development of strategies to block this common pathway, specially the inflammatory pathway, remains a clear unmet clinical need.

It has been well established that CKD is associated with both systemic and local renal inflammation with the participation of crucial inflammatory cells, molecules and pathways, such as macrophages, the nuclear transcription factor-kappa B (NF-κB), the janus kinase/signal transducers and activators of transcription (JAK/STAT) pathway, and inflammatory cytokines [1,2]. Therefore, anti-inflammatory targets directed at specific molecular signatures can be promising therapeutic strategies for CKD. Therapies that target inflammatory pathways at different molecules including pentoxifylline [3], baricitinib [4], NOX-E36 [5], CCX140-B [6], CTP-499 [7] and et al., are on the way. Not surprising, from work with animal models and the overall clinical experiences, it is impossible to reduce renal inflammatory reaction sufficiently by inhibiting a single inflammatory factor. Thus, one attractive therapeutic approach is to target key genes that regulate both immune response and inflammation pathways in CKD. By inhibiting such a molecular target, a global inhibitory effect on renal inflammation will be produced and result in anti-fibrotic effect.

The RNA-binding protein (RBP) Hu antigen R (HuR), also known as embryonic lethal abnormal vision-like protein 1 (ELAVL1), is a ubiquitously expressed post-transcriptional regulator [8]. It has been shown that HuR may be one such protein that controls mRNA turnover and translation of numerous genes involved in immune response, inflammation, fibrosis, and oncogenic signaling pathways, such as COX-2, TNF-a, CCL2, IL-8, TGF-β, VEGF, CyclinD1, and Bcl2 [9-12]. Intracellular HuR is predominantly localized within the nucleus of resting cells. Under various stimuli, HuR binds to the adenine- and uridine-rich elements (AREs) located in 3′-untranslated region (3′-UTR) of mRNA and transports mRNA to cytoplasm to block endonucleolytic cleavage sites, thus protecting mRNA from rapid degradation. After completing the process of stabilizing mRNA, HuR releases itself from the mRNA and returns rapidly to the nucleus [13]. It has been shown that both the nuclear transcription of HuR and HuR nucleocytoplasmic transporting are activated in response to inflammatory signals to stabilize inflammatory mediators [14-17]. Importantly, most pro-inflammatory transcripts contain conserved or semi-conserved AREs in their 3′-UTR [18]. The enhanced HuR/pro-inflammatory factors circuit should be a crucial and specific mechanism for the initiation and maintenance of the inflammatory phenotype observed in tissue inflammation including CKD. In fact, abnormal elevation and cytoplasmic localization of HuR have been shown in varied kidney diseases such as diabetic nephropathy [19,20], hypertension-related nephropathy [21,22], renal malignancies [23], and ischemia-reperfusion-induced kidney injury [24]. Thus, targeting HuR might provide us with an ideal way to against renal inflammation and thereby controlling CKD progression.

Recently, we have discovered a series of small molecule HuR inhibitors at nM to sub-μM *K*_*i*_ values, which dose-dependently inhibit the action of HuR by specifically disrupting HuR–ARE interaction [25,26]. The lead compound KH-3 exhibits superior potency in disrupting HuR–ARE interaction [27]. It has been shown that KH3 selectively inhibited the viability of cancer cells that had high levels of HuR but had no effect on normal cell line *in vitro*. Treatment with KH-3 *in vivo* inhibited breast tumor growth comparing to that of vehicle control. Furthermore, the inhibitory capability of KH-3 on cancer cells was attenuated by HuR stable knockdown with the lentiviral shRNA, indicating the target selectivity of KH-3 [27]. Importantly, inhibition of HuR with KH-3 yielded a significant reduced in the progression of pathological cardiac hypertrophy in a transverse aortic constriction model, as evidenced by a reduction in hypertrophy, dilation and fibrosis, and preserved cardiac function [28]. We hypothesized that treatment with a HuR small molecule inhibitor, KH-3, would inhibit HuR-targeted inflammatory transcripts and inflammatory reactions thereby improving glomerulosclerosis in experimental nephritis rat model, where glomerular HuR is elevated and activated.

## Materials and methods

### Reagents

The HuR inhibitor KH-3 and the inactive analog KH-3B were synthesized as previously described [25,27]. KH-3 and KH-3B powder were dissolved in DMSO at 20 mM as stock solutions for *in vitro* assays. KH3 powder was dissolved in PBS with 5% ethanol and 5% Tween-80 for animal studies [27]. The monoclonal anti-Thy 1.1 antibody, OX-7, was obtained from NCCC, Biovest International, Inc., Minneapolis, MN, U.S.A. Unless specified otherwise, all other reagents were purchased from Sigma Chemical Co. (St. Louis, Missouri, U.S.A.).

### Animals

Experiments were performed on male Sprague Dawley rats (180–200 g) obtained from the SASCO colony of Charles River Laboratories (Wilmington, MA, U.S.A.). Animal maintenance and study procedures described herein were performed at Dr Huang’s Laboratory in University of Utah, according the Public Health Service Policy on Use of Laboratory Animals and were approved by the Animal Care Committee of the University of Utah. Glomerulonephritis was induced by one-time tail vein injection of 1.875 mg/kg of the monoclonal anti-Thy 1.1 antibody, OX-7. OX-7 binds to a Thy 1.1-like epitope on the surface of mesangial cells, causing immune-mediated, complement-dependent cell lysis followed by exuberant matrix synthesis and deposition. Normal control animals were injected with the same volume of PBS [29].

### Experimental design

Three groups of five rats each were assigned and treated as normal control, disease control, and nephritic rats treated with KH-3 at doses of 50 mg · kg^−1^ · day^−1^ based on the preliminary pharmacokinetics of KH-3 (data not shown). KH-3 was administrated by daily intraperitoneal injection from day 1 (24 h after OX-7 injection) to day 5. Untreated normal control and nephritic rats received the daily IP injection of buffer only, served as controls. The urinary protein excretion was measured by the Bradford method (Bio-Rad Protein Assay; Bio-Rad Laboratories Inc. Hercules, CA, U.S.A.) on urine collected from rats housed in metabolic cages for 24 h from day 5 to day 6 after OX-7 injection.

On day 6, all animals were killed under isoflurane anesthesia. Five rats in each group were used for collection of samples. Five to ten milliliters of blood was drawn from the lower abdominal aorta and kidneys were perfused with 30 ml ice-cold PBS. For histological examination, cortical tissue was snap frozen for frozen sectioning or fixed in 10% neutral-buffered formalin for periodic acid-Schiff (PAS) staining. Additional two pieces of renal cortex tissue were quickly frozen in liquid nitrogen and stored at −70°C until analysis of mRNA and protein expression by Western blotting and real-time RT-PCR.

### Determination of liver and renal function

Plasma alanine aminotransaminase (ALT) activity levels were determined using the Heska Element DC Chemistry Analyzer (Heska Fuji Film Corporation, Loverland, CL, U.S.A.). Plasma BUN concentrations were measured by using the QuantiChrom™ urea assay kit.

### Histological analyses

All microscopic examinations were performed in a blinded fashion. Three-micrometer sections of paraffin-embedded tissues were stained with HuR or PAS. The PAS-positive glomerular extracellular matrix was quantitated in a blinded fashion by a computer-assisted color image analysis system as previously described [29]. At least 20 glomeruli from each individual rat were assessed under high magnification (×400). The PAS-positive material area in the mesangium was normalized by that of the total glomerular tuft where the percentage of mesangial matrix occupying each glomerulus area was rated. The average glomerular sclerosis was obtained by averaging scores from all glomeruli on one section.

Indirect immunofluorescent staining for HuR was performed on paraffin-embedded kidney tissues. At the same time, FITC-conjugated wheat germ agglutinin (WGA) (Invitrogen, Carlsbad, CA, U.S.A.) was used to counterstain the glomeruli and tubules to define the location of HuR-positive cells in the kidney [30]. Briefly, deparaffinized and rehydrated kidney sections obtained as above were treated with Dako target retrieval solution (Dako North America, InC., Carpinteria, CA, U.S.A.) for antigen retrieval. The sections were then first stained with FITC-conjugated WGA for half hour at room temperature. Second, the WGA-stained sections were continuously incubated with monoclonal mouse anti-HuR IgG (Santa Cruz Biotechnology Inc. Santa Cruz, CA, U.S.A.) as the primary antibody at 4°C overnight. Alexa Fluor Plus 594-conjugated goat anti-mouse IgG (H+L) (Invitrogen) was applied as the secondary antibody at room temperature for 1 h. Control slides treated with antibody diluent instead of primary antibody showed no staining. After staining, one drop of DAPI-Fluoromount-G was applied on the section to stain the nuclei DNA.

To observe HuR expression in varied glomerular diseases, human renal tissue was obtained for HuR staining from archived paraffin-embedded biopsy material kept at the Department of Internal Medicine, Division of Nephrology, Jiangsu Province Hospital affiliated with Nanjing Medical University. Briefly, three renal sections were obtained from three different normal part of surgically removed tumor kidneys, served as normal controls. Twelve initial renal biopsy sections were obtained from 12 CKD patients with diabetic nephropathy (DN, *n*=3), or lupus nephritis (LN, *n*=3), or diffuse mesangioproliferative glomerulonephritis (MPGN, *n*=2), or IgA-induced focal segmental glomerulosclerosis (IgA-FSGS, *n*=2), or membranous nephropathy (MN, *n*=2). In addition, to briefly assess the potential associations of glomerular HuR levels with glomerular extracellular matrix (ECM) accumulation, 11 initial renal sections were obtained from IgA patients with different extent of glomerular mesangial proliferation, who received a diagnosis of mesangial proliferative IgA nephropathy without any treatment. Three or four-μm-thick renal sections were stained for PAS and HuR described as above and carried out in Nanjing Medical University. Use of the human material was approved by the local ethical review board.

Immunofluorescent staining for fibronectin-EDA+ (FN), type IV collagen (Col-IV), macrophages, Wilms tumor protein 1 (WT-1), and podocin was performed on frozen renal sections and evaluated in 20 glomeruli from each rat as described [29,31]. Briefly, samples were fixed with 4% paraformaldehyde (PFA) for 10 min and permeabilized with 0.1% triton X-100 (Thermo-fisher scientific, Waltham, MA, U.S.A.) for 10 min. Non-specific binding was blocked in PBS containing 10% serum (Vector Laboratories, Burlingame, U.S.A.), for 1 h at room temperature. Primary antibodies of mouse anti-fibronectin-EDA+ (FN) IgG (Harlan Sera-Lab Ltd., Loughborough, U.K.), rabbit anti-type IV collagen (Col-IV) (Rockland Immunochemicals Inc., Limerick, PA) mouse monoclonal anti-CD68 (ED-1) antibody (Abcam, Cambridge, MA, U.S.A.), mouse monoclonal anti-CD163 (ED-2) IgG (Bio-Rad-antibodies.com), rabbit anti-WT-1 IgG (C-19), and goat anti-podocin IgG (G-20) (Santa Cruz) were applied at 4°C for overnight. Either the rhodamine-Red™-X-conjugated donkey anti-mouse IgG or the FTIC-conjugated donkey anti-rabbit or the Cy™3-conjugated goat anti-rabbit IgG, or the rhodamine-Red™-X-conjugated donkey anti-goat IgG (Jackson ImmunoResearch Laboratories Inc.) was used as the secondary antibody. Intraglomerular positive staining of FN, Col-IV, ED-1, ED-2, WT-1 and podocin in rats, and HuR in human kidney tissue was quantified separately in a blinded fashion using image-J (National Institutes of Health, Bethesda, Maryland, U.S.A.) as described previously [29,31].

### Western blot analysis

Renal cortex tissue (15 mg) from each rat was homogenized in lysis buffer (Cell Signaling Technology, Inc., Beverly, MA, U.S.A.) with 1% NP40, 1 mM PMSF, and 1 tablet/5 ml protease inhibitor mix (Complete, Mini; Roche Diagnostics Corporation, Indianapolis, IN). Protein sample at the same amount from each rat in a group was pooled to represent the individual group for further examination. For Western blot analysis, protein samples (20 μg each) were subjected to SDS-PAGE in 4–12% gradient gel (Invitrogen) and immunoblotting on immobilon-P transfer membranes (Millipore Corporation, Bedford, MA, U.S.A.). Proteins of HuR, transforming growth factor-β1 (TGF-β1), plasminogen activator inhibitor-1 (PAI-1), FN, Col-I, Col-IV, podocin, desmin, nuclear factor kappa-B p65 subunit (NF-κB-p65), NAPDH oxidase Nox4, β-actin, and GAPDH were assessed on the Western blots, and the immunostaining band was visualized and quantitated as described previously [31-33].

### RNA preparation and real-time RT-PCR assay

Total RNA was extracted from renal cortex tissue using Tri Reagent according to the manufacturer’s instructions. Two micrograms of total RNA was reverse-transcribed using the superscript III first-strand synthesis system for RT-PCR kit (Invitrogen). Real-time RT-PCR was performed using the SYBR green dye I (Applied Biosystems, Foster City, CA, U.S.A.) and the ABI 7900 Sequence Detection System (Applied Biosystems) as described previously [34]. Samples were run as triplicates in separate tubes to permit quantification of the target gene normalized to β-actin. Sequences of primers used for fibrotic markers such as TGFβ1, PAI-1, FN, Col-I, Col-III, Col-IV, and GAPDH were described previously [31,35].

### Statistical analysis

All data are expressed as mean ± SD. Statistical analyses of differences between the groups were performed by ANOVA and subsequent Student–Newman–Keuls or Dunnett testing for multiple comparisons. Comparisons with a *P* value < 0.05 were considered significantly different. The disease-induced increase in a variable was defined as the mean value for the disease control group minus the mean value of the normal control group (100%). The percent reduction in disease severity in a KH-3-treated group was calculated as follows:

$$\left\{1-\frac{\left(\text{KH-3-treated group mean}\;-\;\text{Normal control group mean}\right)}{\left(\text{Disease control group mean}\;-\;\text{Normal control group mean}\right)}\times 100\right\}$$

## Results

### Glomerular staining and protein expression of HuR in anti-Thy 1.1 nephritis

**Figure 1.**
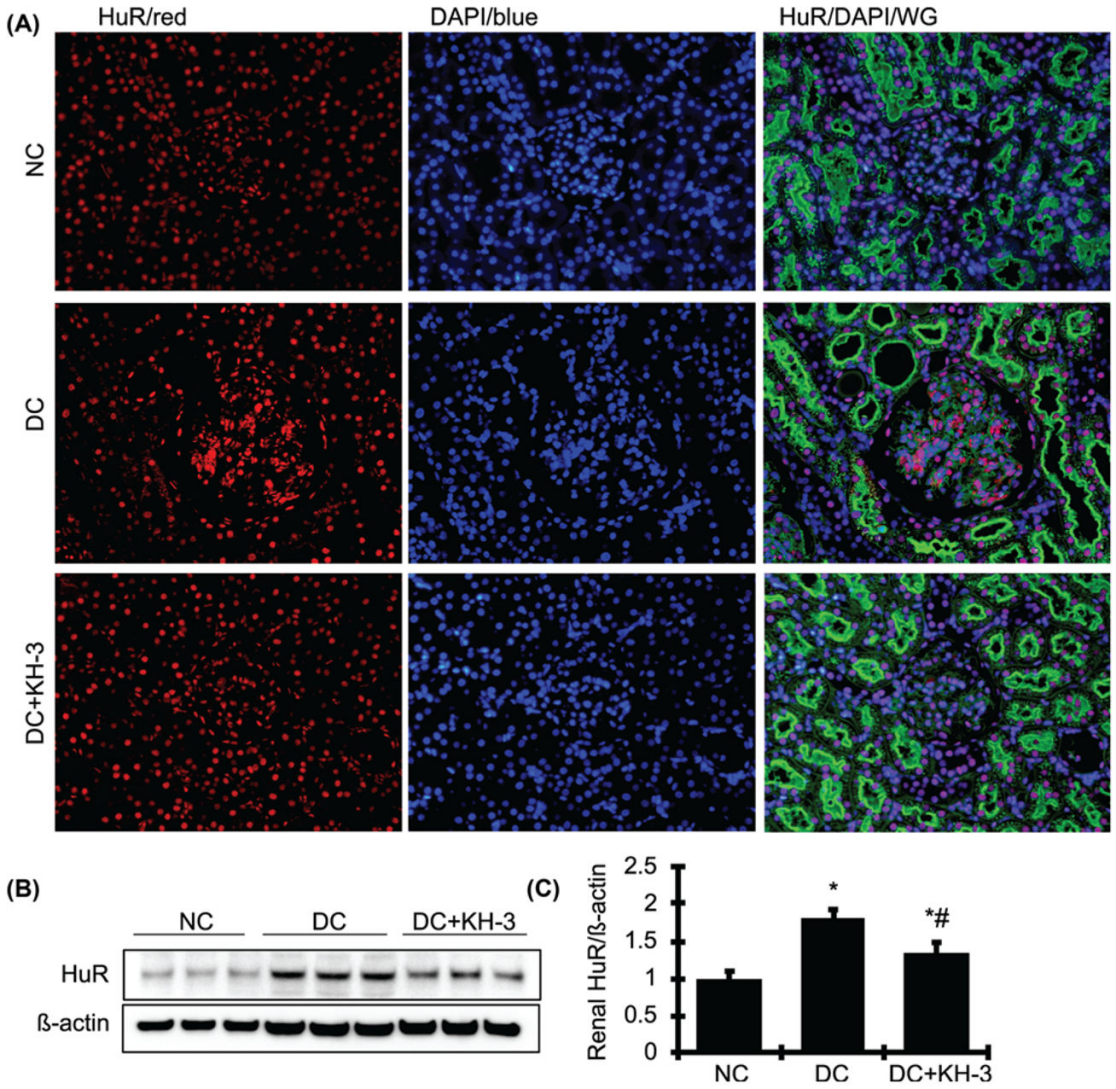
HuR is aberrantly increased and activated in anti-Thy 1.1 nephritis (**A**) Representative photomicrographs of glomerular immunofluorescent staining for HuR (red), DAPI (blue) and wheat germ agglutinin (WGA) (green) (×200 magnification) from the normal rats (NC), diseased rats (DC) treated without or with KH-3 (DC+KH-3). (**B**) Representative Western blots illustrated protein expression of HuR and β-actin in the renal cortex tissue (*n*=5/each group). (**C**) Quantification of the band density is shown on the right of Western blot. Protein values are expressed relative to normal control, which was set at unity. KH-3 treatment resulted in a significant reduction in glomerular HuR staining and protein production levels compared with diseased control rats; *P<0.05, vs. NC; #*P*<0.05, vs. DC.

As shown in Figure 1A, HuR was stained in red color inside of nuclei of all glomerular and tubular cells and no cytoplasm staining of HuR was observed in normal rats. In disease control rats, glomerular cell number was significantly increased, and those cells were all HuR-positive. Furthermore, the staining intensity of nuclear HuR in those cells was enhanced and the cytoplasm staining of HuR was also observed in some glomerular cells. Glomeruli from KH-3-treated rats had less cell number and less staining intensity of HuR, compared with those disease controls. However, a few glomerular cells still showed the positive staining of cytoplasm HuR. No change in staining pattern or intensity was observed in tubular cells in these three groups of rats. As further verification of these staining results, the HuR protein abundance from renal cortex tissue was determined by Western blot assay (Figure 1B). The amount of HuR protein was increased by 1.82-fold in disease control rats, compared with normal rats, which was partially reduced by KH-3 treatment (Figure 1C). These data indicate that of all kidney tissue, glomerular cells effect the greatest changes in HuR abundance and cytoplasmic translocation, consistent with the injury only induced in glomeruli in this model.

### Therapeutic efficacy of KH-3 for renal fibrosis

#### KH-3 safety and its effects on renal function and urinary protein excretion in anti-Thy 1.1 nephritis

**Figure 2.**
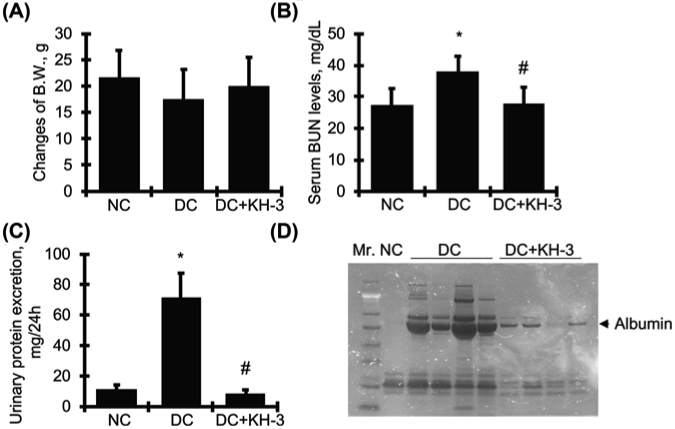
Effect of KH-3 on body weight, renal function, and proteinuria in nephritic rats (**A**) Changes of rat body weight before and after treatment with KH-3 (*n*=5/each group). (**B**) Changes of renal function determined by serum BUN levels (*n*=5/each group). Serum BUN levels were significantly reduced in the KH-3-treated nephritic rats. (**C**) Urinary protein excretion was significantly lower in the KH-3-treated, nephritic group. *P<0.05, vs. normal control (NC); #*P*<0.05, vs. diseased control (DC). DC+KH-3, diseased rats treated with KH-3. (**D**) SDS-PAGE electrophoresis of 24-h urine samples. Mr. protein markers. One thousandth of pooled 24-h urine sample of normal rats (NC, *n*=5) or individual 24-h urine sample from diseased rats (DC, *n*=4) or KH-3-treated diseased rats (DC+KH3, *n*=4) was run and dyed on SDS-PAGE gel. Arrow indicates albumin protein.

The final clinical parameters for the three groups of rats are shown in Figure 2. There was no difference in daily food intake, water intake, urine excretion, and body weight among these three groups (Figure 2A). By monitoring side effects of KH-3, we also observed that none of treated rats had diarrhea or peritonitis. Furthermore, plasma hepatic injury marker ALT activity levels were in normal range, less than 10 U/l in all treated rats, which were the same as those in normal control rats or untreated disease rats. These data indicate that KH-3 is a safe reagent to be given to rats *in vivo*. However, those diseased rats had increased serum urea levels (*P*<0.05) compared with normal control rats. The elevated serum urea levels were reduced to normal level by KH-3 treatment (Figure 2B).

Twenty-four-hours of urinary protein excretion was measured from day 5 to day 6 (Figure 2C). Disease-induced increases in urinary protein excretion were markedly reduced by KH-3 treatment compared with the disease control group, which was easily visualized by the electrophoresis of SDS-PAGE gel of urine samples in Figure 2D.

#### Effects of KH-3 on glomerular matrix accumulation in anti-Thy 1.1 nephritis

**Figure 3.**
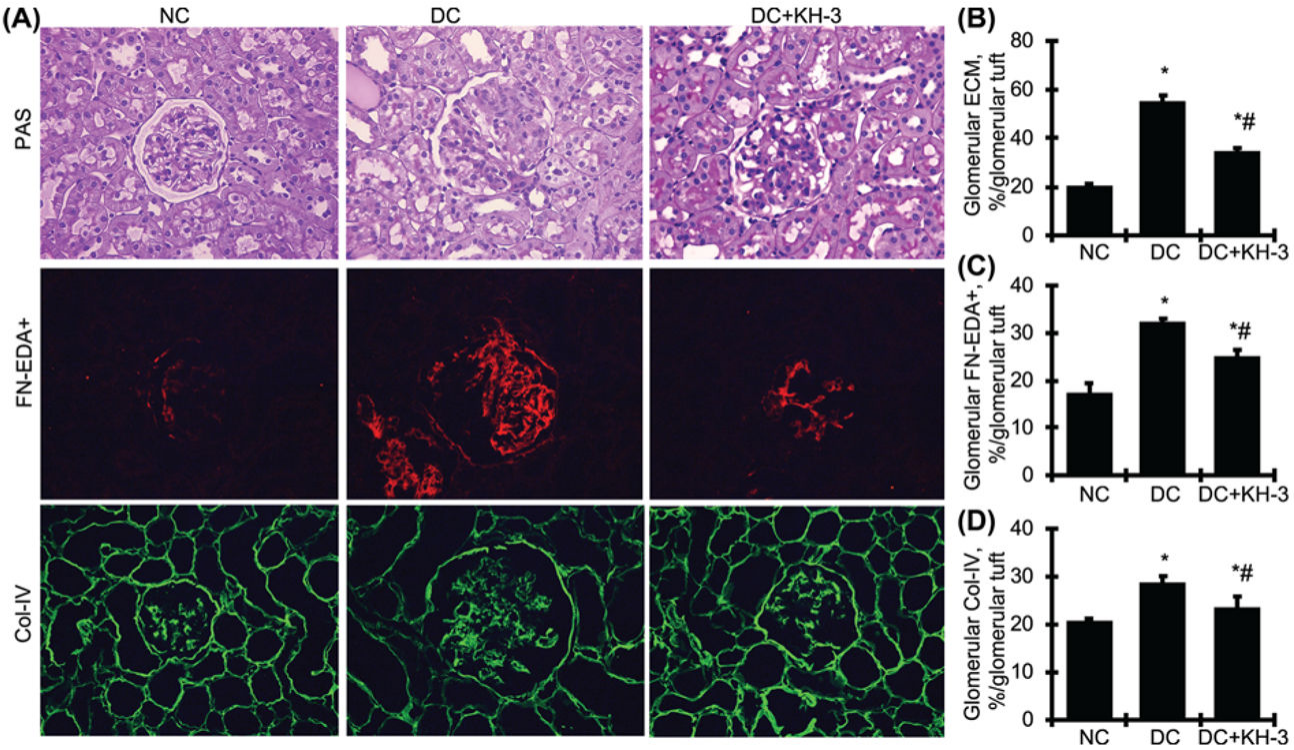
KH-3 treatment reduces glomerulosclerosis in nephritic rats (**A**) The histological renal sections stained with PAS or immunofluorescent stained for FN and Col-IV are presented at 200× magnification. Graphic representation of glomerular staining score for ECM (**B**), FN (**C**), and Col-IV (**D**) is shown on the right, respectively. KH-3 treatment resulted in a significant reduction in glomerular PAS staining score, FN and Col-IV staining score compared with diseased control rats. *P<0.05, vs. normal control (NC); #*P*<0.05, vs. diseased control (DC). DC+KH-3, diseased rats treated with KH-3; *n*=5/each group.

Representative glomeruli stained with PAS, FN, and Col-IV are shown in Figure 3A. The untreated diseased rats developed glomerular fibrosis with a marked increase in glomerular extracellular mesangial matrix, stained pink with PAS, including matrix components measured by immunofluorescent staining for FN and Col-IV at day 6, compared with normal rats (Figure 3A). These lesions in glomerular area were markedly reduced in KH-3-treated rats. Figure 3B-D shows the graphical representations of the mean ± SD of each glomerular staining score for each group. Glomerular PAS, FN, and Col-IV staining scores increased by 268%, 184%, and 139% respectively in disease control rats as a result of nephritis, compared with normal control rats. KH-3 treatment decreased the matrix score significantly, which are shown by the decreases in disease-induced ECM accumulation of 59% for PAS (Figure 3B), 49% for FN (Figure 3C), and 64% for Col-IV (Figure 3D).

#### Effects of KH-3 on renal mRNA and protein levels of TGFβ1, PAI-1, FN, and collagen in anti-Thy 1.1 nephritis

**Figure 4.**
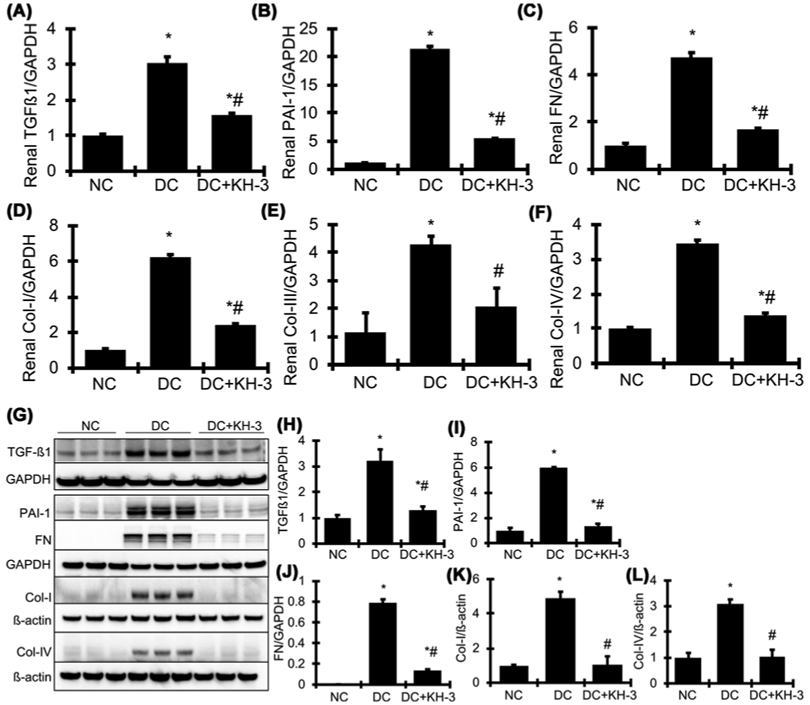
KH-3 treatment reduces renal mRNA expression and protein production of profibrotic markers in nephritic rats (**A–F**) Expression of TGFβ1 (A), PAI-1 (B), FN (C), Col-I (D), Col-III (E), and Col-IV (F) mRNA was determined by real-time RT/PCR. Changes in mRNA levels were determined by first correcting the amplification of GAPDH for each sample. (**G**) Representative Western blots illustrating TGFβ1, PAI-1, FN, Col-I, Col-IV, GAPDH, and β-actin protein expression in renal cortical tissues. (**H–L**) The graphs summarize the results of band density measurements for those protein markers. *P<0.05, vs. normal control (NC); #*P*<0.05, vs. diseased control (DC). DC+KH-3, diseased rats treated with KH-3; *n*=5/each group.

As two key modulators of matrix accumulation, TGFβ1, and PAI-1 play an important role in renal fibrosis. Of note, both TGFβ1 and PAI-1 were also found to be the putative target of HuR in human cancer cells since both of them contain conserved AREs in their 3′-UTR [17,21]. As shown in Figure 4A-F, renal mRNA analysis revealed a 3-fold increase in TGFβ1 mRNA expression and dramatic increases in PAI-1 mRNA expression in disease control rats compared with normal rats. Consequently, renal FN, Col-I, Col-III, and Col-IV mRNA levels were increased by 4.74-, 6.23-, 4.26-, and 3.45-fold respectively in disease control rats. KH-3 administration significantly reduced the levels of renal TGFβ1, PAI-1, FN, Col-I, Col-III, and Col-IV mRNAs, by 71.7%, 78.2%, 81.5%, 73%, 67.5%, and 84.5%, respectively. In agreement with the increased mRNA expression levels, the protein levels of TGFβ1, PAI-1, FN, Col-I, and Col-IV in renal cortex tissue further quantified by Western blot assay were markedly increased in disease control rats, compared with normal rats (Figure 4G-L). However, the protein production levels of these fibrotic markers in renal cortex were reduced significantly with KH-3 treatment.

#### Effects of KH-3 on podocyte injury in anti-Thy 1.1 nephritis

Although the primary injury, i.e., injection of a complement-activating anti-Thy 1.1 antibody, OX-7, is highly selective for renal mesangial cells, intraglomerular capillaries are destabilized in the course of mesangial cell destruction, leading to capillary dilation and intraglomerular microaneurysms after disease induction [36]. All of these changes contribute to secondary podocyte damage via altered physical forces and/or potential biochemical alterations of the glomerular basement membrane [37,38], which may promote proteinuria and glomerular fibrosis in anti-Thy 1.1 nephritis.

**Figure 5.**
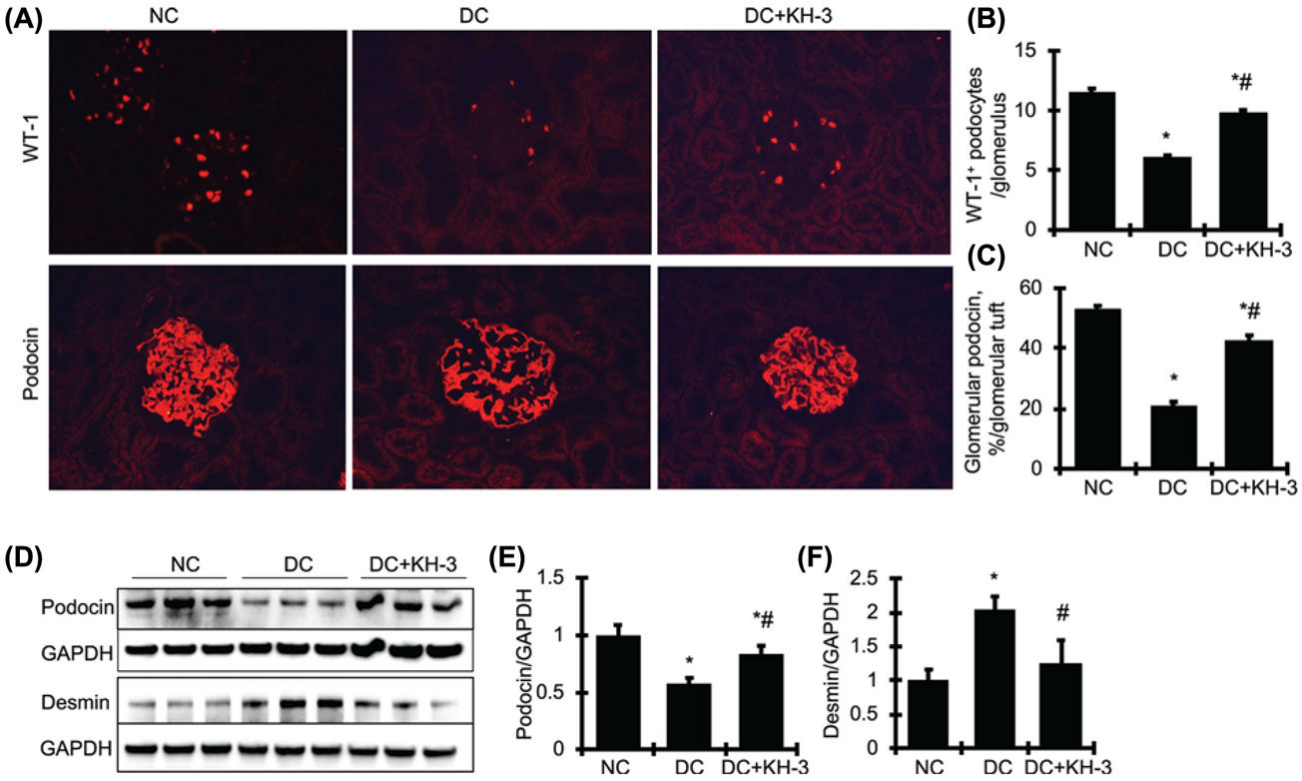
KH-3 treatment ameliorates podocyte markers in nephritic rats (**A**) The immunofluorescent staining of renal sections for glomerular WT-1 and podocin; 200× magnification. (**B** and **C**) Graphic representations of glomerular staining number of WT-1 positive podocytes (B) or score of podocin (C) are shown on the right. (**D**) Representative Western blots illustrating, podocin, desmin, and GAPDH protein expression in renal cortical tissues. (**E** and **F**) The graphs summarize the results of band density measurements shown on the right of the blots. *P<0.05, vs. normal control (NC); #*P*<0.05, vs. diseased control (DC). DC+KH-3, diseased rats treated with KH-3; *n*=5/each group.

As shown in Figure 5A detected by immunofluorescent staining for glomerular podocytes, nephritic glomeruli from disease control rats contained much fewer WT-1 positive podocytes than did glomeruli from normal control rats. The average number of podocytes per glomerular cross-section in nephritic rats treated with KH-3 was 67% higher than that in disease controls (Figure 5B). In addition, diminished staining of podocin (Figure 5A), a critical component of the filtration slits of podocytes [39], was observed in nephritic glomeruli on day 6 as previously [31]. However, staining intensities for podocin were effectively restored after KH-3 treatment. The results of the quantitative analysis of immunofluorescent staining for podocin are shown on the right of Figure 5C.

By Western blot analyses of the renal tissue from different groups of rats, we also observed that the protein levels of podocin in nephritic glomeruli were substantially down-regulated by 42%, compared with normal glomeruli. KH-3 treatment significantly reversed the decreased podocin protein level by 25.4% (Figure 5D,E). Protein levels of desmin, a conventional marker of podocyte injury, were up-regulated by 203% in nephritic kidneys (Figure 5F). Although elevated production of desmin was not specifically confirmed in podocytes, the changes of desmin were consistent with the change of the slit-diaphragm-associated protein podocin, suggesting nephritic rats sustain injury to podocytes. Consistent with improvement of podocyte numbers and podocin levels, KH-3 treatment also successfully reduced the elevated desmin protein expression in nephritic rats by 75.6%.

#### Effects of KH-3 on glomerular monocyte/macrophage infiltration, macrophage polarization and related inflammatory factor expression in anti-Thy 1.1 nephritis

**Figure 6.**
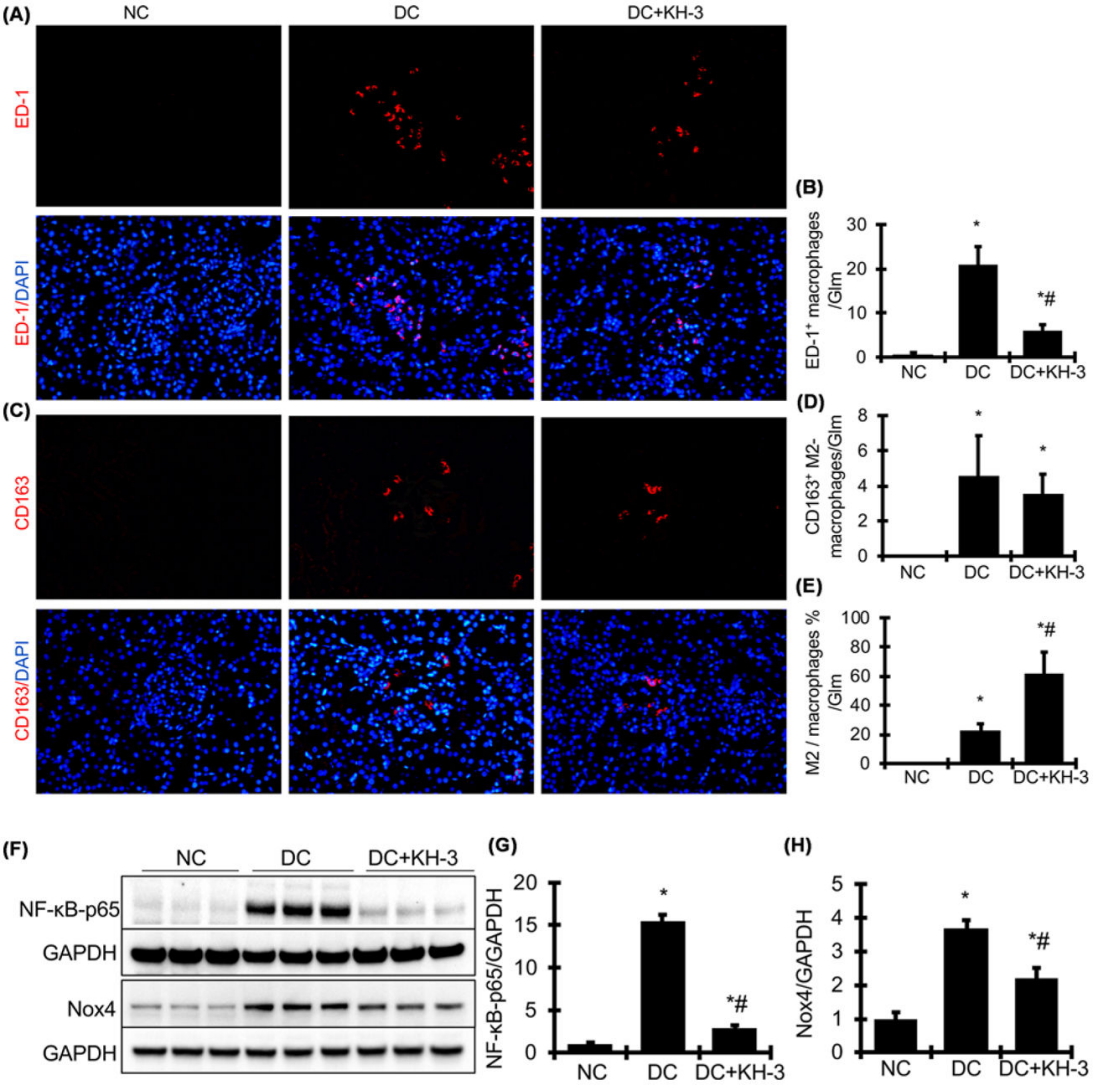
KH-3 treatment reduces number of monocytes/macrophages infiltrating glomeruli, induces M2 macrophage polarization and reduces renal production of NF-κB-p65 and Nox4 in nephritic rats (**A–E**) The immunofluorescent staining of renal sections for ED-1 positive macrophages (A) or CD163 positive M2-macrophages (C) (200× magnification). Graphic representation of glomerular number of macrophages (B), or M2 macrophages (D), or the ratio of subsets of M2-macrophages within glomeruli (Glm) (E) is shown on the right respectively. (**F**) Representative Western blots illustrating, NF-κB-p65, Nox4 and GAPDH protein expression in renal cortical tissues. (**G** and **H**) The graphs summarize the results of band density measurements for NF-κB-p65 (G) and Nox4 (H), respectively. *P<0.05, vs. normal control (NC); #*P*<0.05, vs. diseased control (DC). DC+KH-3, diseased rats treated with KH-3; *n*=5/each group.

The number of monocytes/macrophages was determined in kidney sections from all rats in each group. As shown in Figure 6A, the majority of normal rat glomeruli had no macrophages or one detectable monocyte/macrophage. However, nephritic glomeruli from disease control rats contained much higher numbers of monocytes/macrophages than did glomeruli from normal control rats, which was reduced by 72.8% in the KH-3 treated nephritic rats (Figure 6A,B). Furthermore, although we observed that diseased glomeruli from disease control rats contained similar number of subsets of CD163-positive M2-type macrophages (since CD163 is often used as a highly specific marker of M2-type macrophages [40]) (Figure 6C) when compared with the KH3-treated nephritic glomeruli (4.54±2.30 vs. 3.58 ± 1.08, *P*>0.05) (Figure 6D), the percentage of subsets of M2-type macrophages in untreated diseased glomeruli was much less than that in KH-3 treated glomeruli (22.4% vs. 62.0%, *P*<0.05) (Figure 6E). Similar to ED-1 positive macrophages, there was no detectable M2-type macrophages in normal rat glomeruli (Figure 6C).

We then tested renal pro-inflammatory factor, NF-κB-p65 by Western blot analysis and the results revealed an increased renal production of NF-κB-p65 in disease control rats compared with normal rats, which was lowered dramatically by KH-3 treatment (Figure 6F-H). Furthermore, renal protein levels of Nox4, the family member of NAPDH oxidase, were much greater in disease control rats compared with normal controls (Figure 6F,H), indicating significant renal activation of Nox4 in nephritic rats. However, it was reduced by 55% by KH-3 treatment (Figure 6G,H).

Together, these data indicate that macrophage accumulation in glomeruli, as a direct response to the deposition of antibody and antibody-induced mesangial cell lysis in anti-Thy 1.1 nephritis [41], may mediate generation and activation of NF-κB and Nox4. Treatment with KH-3 inhibits glomerular macrophage infiltration and production of NF-κB and Nox4. The latter may be also partially due to KH-3-induced M2 macrophage polarization since the major phenotype of macrophages is the anti-inflammatory M2 phenotype within glomeruli after KH-3 treatment.

#### Elevated glomerular HuR was detected in varied human glomerular diseases

**Figure 7.**
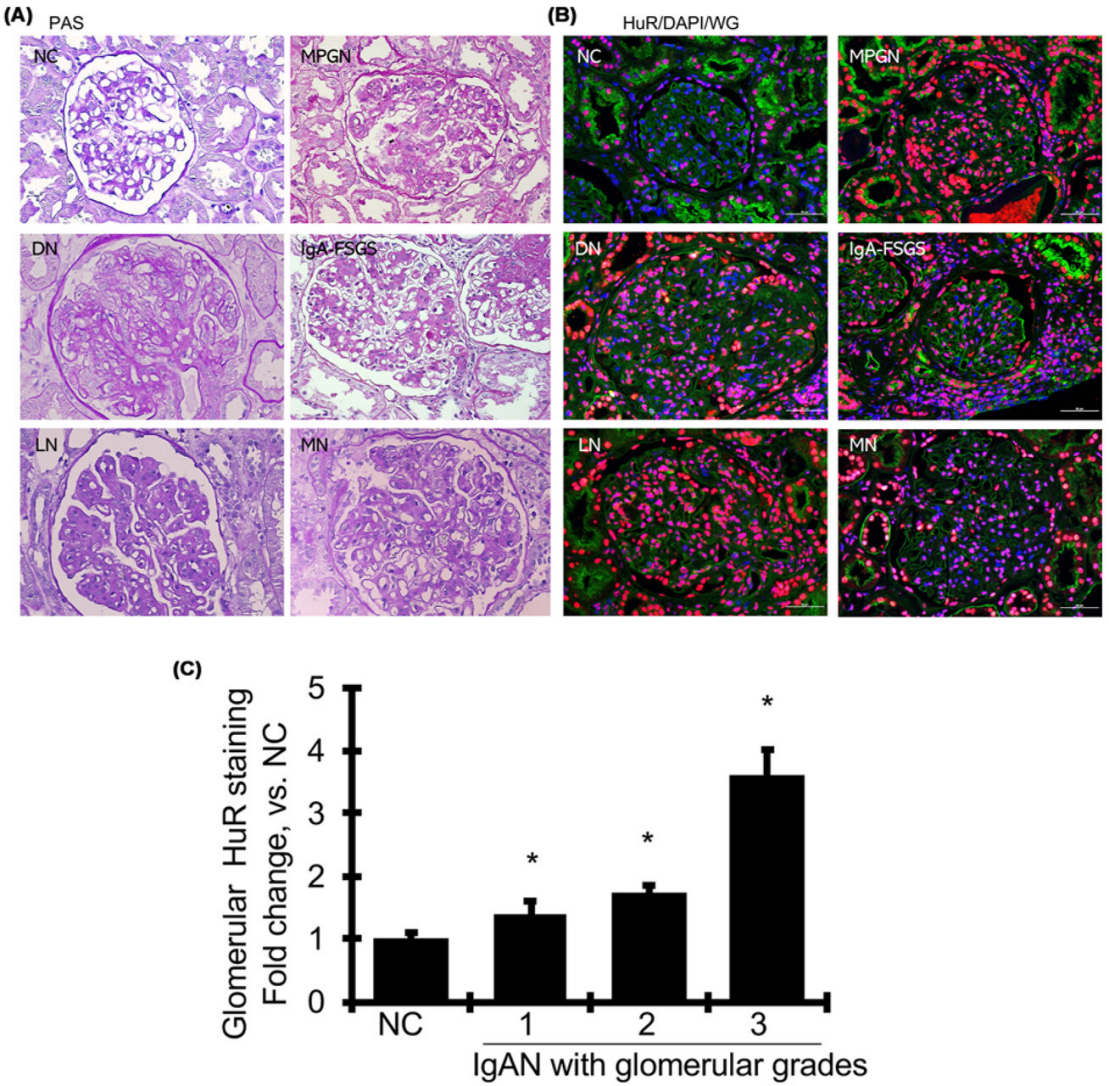
Glomerular HuR expression was elevated in patients with varied glomerular diseases (**A**) Representative microphotographs stained for PAS from kidney biopsy tissue of patients with advanced diabetic nephropathy (DN, *n*=3), or lupus nephritis (LN, *n*=3), or diffuse mesangioproliferative glomerulonephritis (MPGN, *n*=2), or IgA-induced focal segmental glomerulosclerosis (IgA-FSGS, *n*=2), or membranous nephropathy (MN, *n* =2) (400× magnification) and their immunofluorescent stained for HuR (red), DAPI (blue) and wheat germ agglutinin (WGA) (green) (**B**). Scale bars for HuR staining photos represent 50 μm. (**C**) Glomerular HuR staining score levels from IgA nephropathy with different degrees of glomerular sclerosis grades (GG) as described in the results, compared with normal kidneys and expressed as fold increase over normal levels. IgAN, GG-1, *n*=3; GG-2, *n*=4 and GG-3, *n*=4. *P<0.05, vs. normal control (NC, *n*=3).

To investigate whether HuR is also elevated in the kidneys of patients with varied glomerular diseases, we randomly chosen 12 renal biopsy tissues from CKD patients with advanced either DN, or LN, or MPGN, or IgA-FSGS or MN and their PAS-stained pictures were shown in Figure 7A respectively. Interestingly, glomerular HuR staining in all those diseased glomeruli was dramatically increased compared to normal glomeruli, as shown in Figure 7B. In addition, most of those patients already displayed a certain degree of tubular injury and interstitial fibrosis as shown in their PAS staining. Importantly, tubulointerstitial HuR staining was increased in those patients, which is different with that seen in anti-Thy 1.1 nephritic rat model, where there was no tubular injury. Then, we further analyzed 11 initial human renal biopsy tissues with IgA nephropathy to evaluate the possible association of glomerular HuR expression and glomerular sclerosis. Those patients with documented disease exhibited hematuria and proteinuria and different grades of glomerular lesion. Here, glomerular grade (GG) was assessed by PAS staining according to a reported glomerular grading system, which was based solely on the amounts of mesangial matrix extent of segmental sclerosis in each glomerulus [42], such as GG-1, mean sclerosis per glomerulus, 0% to less than 25%; GG-2, 25% to less than 50%; and GG-3, 50% to 100%. Interestingly, both the glomerular cell number positive for HuR staining and the cellular staining intensity of HuR were enhanced in diseased glomeruli with IgA nephropathy from mild to diffuse marked mesangial proliferation and the staining levels of glomerular HuR quantified by Image-J were markedly increased in IgA nephropathy with increased glomerular grading and sclerosis (Figure 7C). The limitation here is that all those HuR staining in human biopsy tissue was carried out on 3-micron, not 1-micron, thick sections, which may not be representative of standard pathological evaluation of human kidney disease according to international evaluation guidelines of human kidney disease by kidney biopsy. Further detail evaluation of HuR staining and expression on human kidney biopsy tissue needs to be determined in the future. Nonetheless, compared with HuR staining in normal kidney tissue, up-regulation and activation of glomerular HuR observed briefly here acting as a feature of glomerular disease in humans may be suggested. Elevated tubulointerstitial HuR seen in humans may be involved in the development or progression of tubulointerstitial fibrosis as well.

## Discussion

In the present study, the anti-Thy 1.1-induced glomerulonephritis rat model resembles disease in many ways seen in human patients, including proteinuria, elevated blood urea levels, visible damage of podocytes on the immunofluorescent staining, renal hypertrophy, and glomerulosclerosis [29,31]. These fibrotic responses are mainly related to increased infiltration of inflammatory cells, up-regulation of pro-inflammatory, oxidative stress-related and pro-fibrotic molecules, as we repeatedly observed in the present study. Importantly, although expressed at lower levels in normal glomeruli, HuR was found to be selectively up-regulated in glomerular residential cells and newly infiltrated or proliferated glomerular cells in nephritic glomeruli in this model. A shift of HuR to the cytoplasm, where it regulates target mRNA stability and translation, was also observed and increased in diseased glomeruli. Those abnormal elevated glomerular HuR abundance and nucleocytoplasmic transporting occurred at the site of injury, found first in the present study, are in consistent with previous findings in other kidney disease models [19,20,23,24] and in kidney biopsies from patients with varied glomerular diseases. Although the mechanism of how HuR expression is up-regulated in kidney disease is largely unknown, the concurrently enhanced mRNA expression and translation of profibrotic factors and proinflammatory factors observed in present study, as the putative targets of HuR, suggest that enhanced HuR function is causally linked to the onset and progression of glomerular nephropathy. Furthermore, diseased rats treated with a small molecule HuR inhibitor, KH-3, showed reduced glomerular HuR generation and activation and reduced disease severity, as evidenced by reduced impaired renal functional and structural changes that were seen in untreated diseased rats. Our study provides compelling evidence for a pivotal role of elevated HuR in renal inflammation and fibrogenic process in kidney diseases. On the other hand, our study also confirms the therapeutic potential of KH-3 for protection of renal function and glomerulosclerosis via inhibition of HuR function and the HuR-regulated genes.

Although we did not screen the mRNA changes of all HuR targets in KH-3 treated nephritis rats, both TGFβ1 and PAI-1 among those HuR targets are well-known factors that play a critical role in the pathogenesis of organ fibrosis through stimulating matrix synthesis and accumulation and the oxidative/inflammatory insults alongside the corresponding activation of multicellular profibrotic signaling pathways. Of note, TGFβ1 itself has been shown to induce HuR cytoplasmic translocation, which in turn increases the mRNA stability thereby further increasing endogenous TGFβ1 expression and secretion [16]. Apparently, inhibition of HuR with KH-3 is able to further disrupt the positive TGFβ1/HuR feedback circuit in glomerular disease. NF-κB is a critical transcription factor involved in a broad range of biological processes. Deregulated NF-κB activity has been observed in various diseases, including chronic inflammation and renal fibrosis [43]. It is unknown yet whether NF-κB is the direct HuR target. However, reduction of TGFβ1, or oxidative stress that has been demonstrated to lead to NF-κB activation should suppress NF-κB’s activation and action. By contrast, NF-κBp65 has been shown to activate HuR transcription during gastric tumorigenesis [44]. It is possible that NF-κBp65 signal activation observed in nephritic glomeruli stimulates glomerular HuR transcription and protein modifications that may drive HuR cytoplasmic translocation, as TGFβ1 does. Very recently, Nox4 was identified as one of critical HuR targets, particularly in diabetic nephropathy [45]. Induced expression of Nox4 contributes to kidney cell injury via generation of reactive oxygen species (ROS) [46]. Interestingly, transduction of adenoviral Nox4 to increase Nox4 generation and activation in hepatic stellate cells also resulted in increased cytoplasmic shuttling of HuR [47]. It is possible that there is another Nox4/HuR feedback circuit regulating the fibrogenic response in nephritic glomeruli, in addition to the TGFβ1/HuR feedback loop and all these effects are inhibited by KH-3 treatment. Collectively, these observations elucidate an important link between cellular HuR activation and signaling of TGFβ1, NF-κB, and Nox4 in glomerular disease. Thus, the beneficial effects of inhibition of HuR-ARE interaction of HuR targets by KH-3 on glomerular inflammation and fibrosis in anti-Thy 1.1 nephritic rats that may be superior than each inhibitor alone of those targeted molecules are easy to interpret.

In the present study, KH-3 treatment actually reduced glomerular HuR protein abundance and partial HuR nucleocytoplasmic transporting (Figure 1). Although not proven here, it is likely that reduced HuR generation and activation lead to further decreases in HuR targeted mRNAs’ stability, translation and action, as we observed. These results would not be expected if the sole action of KH-3 was to disrupt the HuR-ARE interaction of HuR-targets. In fact, previous reports have shown that HuR abundance is auto-regulated by a positive-feedback loop involving HuR interaction with the 3′-UTR of its own mRNA [48]. The authors suggested that HuR mRNA is destabilized through an ARE-dependent but unidentified mechanism. It is likely that KH-3 could potentially disrupt HuR for binding to its own 3′-UTR, reducing HuR mRNA stability, decreasing HuR mRNA abundance and nucleocytoplasmic transporting directly, in addition to the indirect effect of KH-3 on HuR activation via inhibition of TGFβ, NF-κB and Nox4 signaling as described above. Thus, KH-3 treatment is able to further suppress HuR-targeted gene protein expression, thereby further contributing to reduced disease severity of anti-Thy 1.1 nephritis.

It is believed that proteinuria is one of important factors leading to the progression of kidney disease. Inhibition of HuR with KH-3 sharply reduced proteinuria in the present study. The exact mechanisms of this action of KH-3 are unclear. With increasing attention being paid recently to the multiple roles of the podocyte in maintaining glomerular function [38,49,50], inhibition of HuR with KH-3 may have a potential to protect podocytes from being damaged and thereby promoting reduction of proteinuria. Indeed, the podocyte injuries caused by the induction of anti-Thy 1.1 nephritis were evaluated in the present study not only by the amount of albuminuria/proteinuria, but also by the expression of WT-1, podocin, and desmin. The immunohistological staining of podocin and WT-1-positive podocytes and the protein level of podocin dramatically decreased in diseased glomeruli. Podocin is reported to be one of the critical molecules for maintaining the barrier function of the podocyte slit diaphragm [51]. Reduction of podocin may cause the consequent podocyte shape changes, such as the effacement of the foot [51]. In addition, podocyte epithelial–mesenchymal transition (EMT), characterized by the loss of epithelial markers and gain of mesenchymal markers such as desmin, has been also involved in podocyte foot process effacement [52,53]. In the present study, we observed an increase in desmin in nephritic glomeruli. Taken together, it is conceivable that the marked proteinuria in anti-Thy 1.1 glomerulonephritis results from the podocyte dysfunction and even reduction of the podocyte number. However, the podocytic dysregulation of podocin and desmin was counter-regulated when KH-3 was given. Suppression of HuR lowers HuR targets mRNA expression and action in glomerular podocytes may directly impose on podocytes. In addition, there might be a pathogenic cross-talk occurred between mesangial cell (cause) and podocyte (injury) in anti-Thy 1.1 nephritis [50,54]. HuR-targets such as TGFβ1, PAI-1, and Nox4 may act as the messenger of the injury signal from mesangial cells to podocytes in this model. Inhibition of HuR-targeted genes with KH-3 may block this potential cross-talk thereby reducing the response of podocyte to injury. Likely, podocyte protection may explain the reason why therapy with KH-3 markedly decreased albuminuria/proteinuria.

In conclusion, accumulated evidence indicates that local cellular HuR is aberrantly increased and activated in varied kidney diseases in response to varied stimuli including persistent stress and inflammation. The present study further demonstrates that up-regulation of glomerular HuR plays an important role in renal inflammation and fibrogenic process in glomerulosclerosis not only in anti-Thy 1.1 nephritis model but also in varied human glomerular diseases. KH-3, a potent small molecule inhibitor of HuR, that targets to the HuR-AREs interaction in the 3′-UTR of target mRNAs, significantly reduces proteinuria and pathological glomerular ECM expansion by a combination of mechanisms including down-regulating HuR its own abundance and activation, decreasing the generation and action of HuR-targeted glomerular TGFβ1, PAI-1, NF-κB and Nox4, inhibiting inflammatory cell infiltration, M2 macrophage polarization and NAPDH-oxidase-mediated oxidative stress and protecting podocyte from injury. The limitation of the present study is that all protein and mRNA levels were determined in renal cortex tissue, instead of the isolated glomeruli as described previously [29,55,56]. However, all those results are consistent with the histological outcomes, typically in renal glomeruli. Nonetheless, our results together with the therapeutic effect of KH-3 for cardiac fibrosis suggest that investigation of this small molecule KH-3, and other therapeutic agents aimed at regulating HuR abundance and function, may open new avenues for the treatment of renal fibrosis.

## Clinical perspectives

- RNA-binding proteins represent an emerging class of proteins with a role in renal dysfunction.
- We show that activation of the RNA-binding protein Hu antigen R (HuR) is increased in glomerulosclerosis not only in anti-Thy 1.1 nephritis model but also in varied human glomerular diseases.
- KH-3, a potent small molecule inhibitor of HuR, that targets to the HuR–AREs interaction, significantly reduces proteinuria and glomerulosclerosis in experimental nephritis rat model, suggesting that investigation of the small molecule KH-3, and other therapeutic agents aimed at regulating HuR abundance and activation, may open new avenues for the treatment of renal fibrosis.

## Abbreviations

CKD: chronic kidney disease
Col-IV: type IV collagen
ECM: extracellular matrix
ELAVL1: embryonic lethal abnormal vision-like protein 1
EMT: epithelial-mesenchymal transition
ESRD: end-stage renal disease
FN: fibronectin-EDA+
GG: glomerular grade
HuR: human antigen R
JAK/STAT: janus kinase/signal transducers and activators of transcription
NF-κB: nuclear transcription factor-kappa B
PAI-1: plasminogen activator inhibitor-1
RBP: RNA-binding protein
ROS: reactive oxygen species
TGF-β1: transforming growth factor-β1
WT-1: Wilms tumor protein 1
